# Supplementary figures and images for: Lacosamide adjunctive therapy for partial-onset seizures: a meta-analysis
Source: PeerJ. 2013 Aug 6;1:e114. doi: 10.7717/peerj.114 (PMC3740140; doi:10.7717/peerj.114)

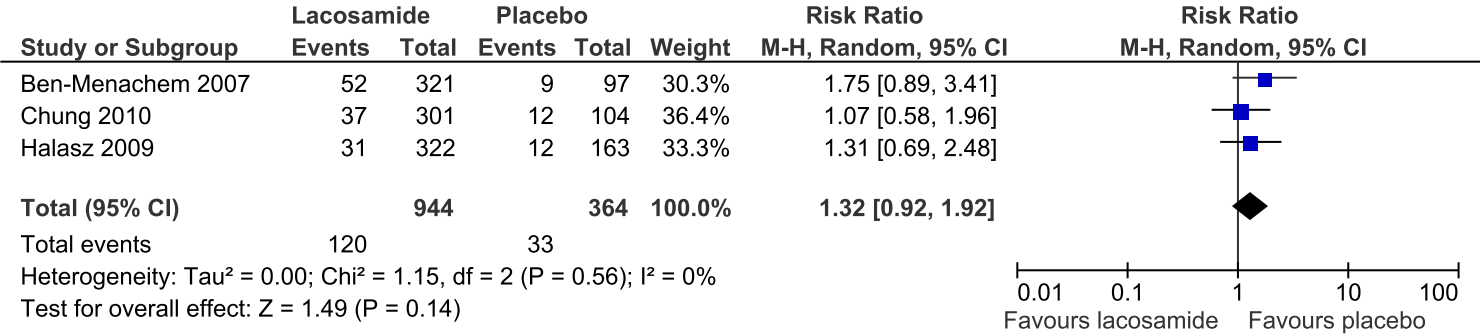

Supplement: Supplemental Information 1 [file peerj-01-114-s001.pdf]

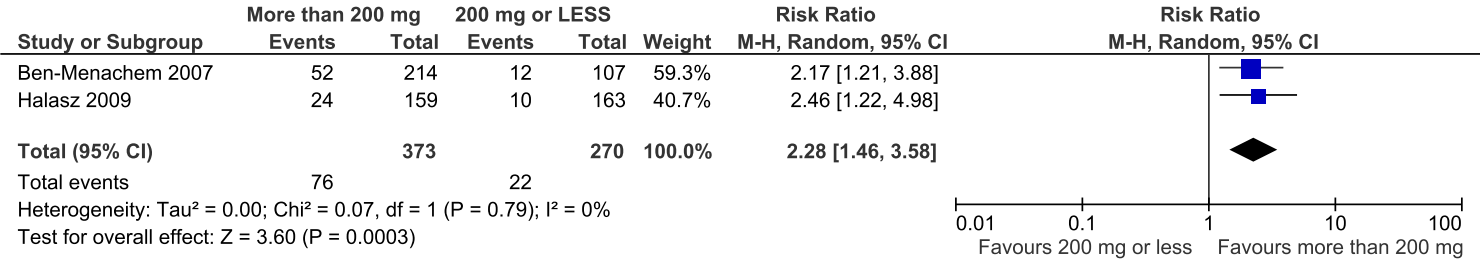

Supplement: Supplemental Information 2 [file peerj-01-114-s002.pdf]

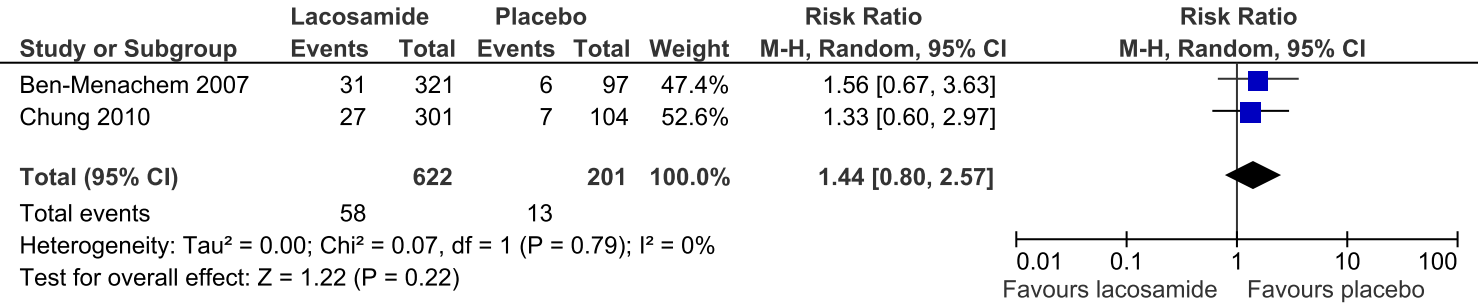

Supplement: Supplemental Information 3 [file peerj-01-114-s003.pdf]

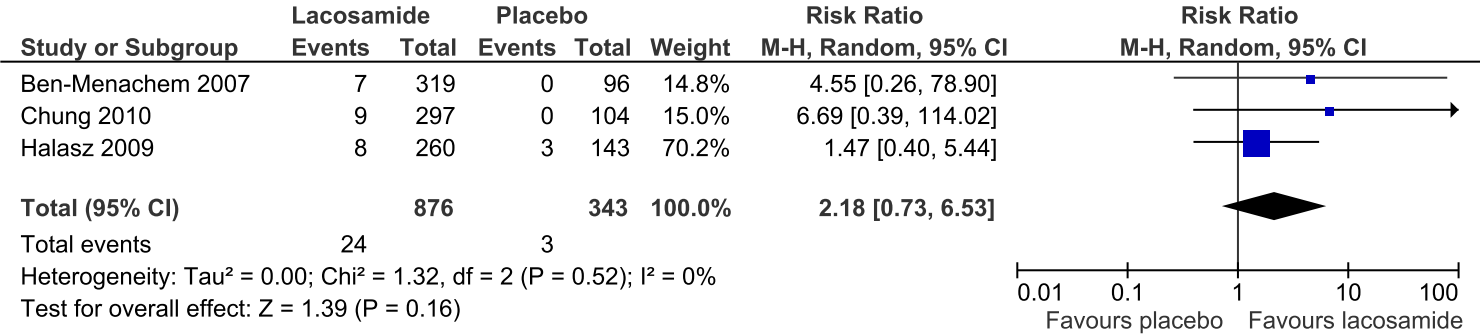

Supplement: Supplemental Information 4 [file peerj-01-114-s004.pdf]

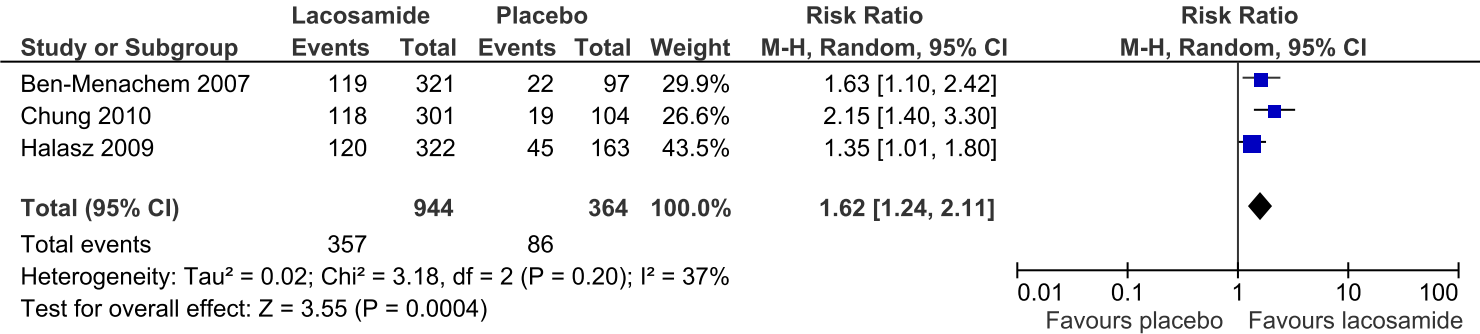

Supplement: Supplemental Information 5 [file peerj-01-114-s005.pdf]

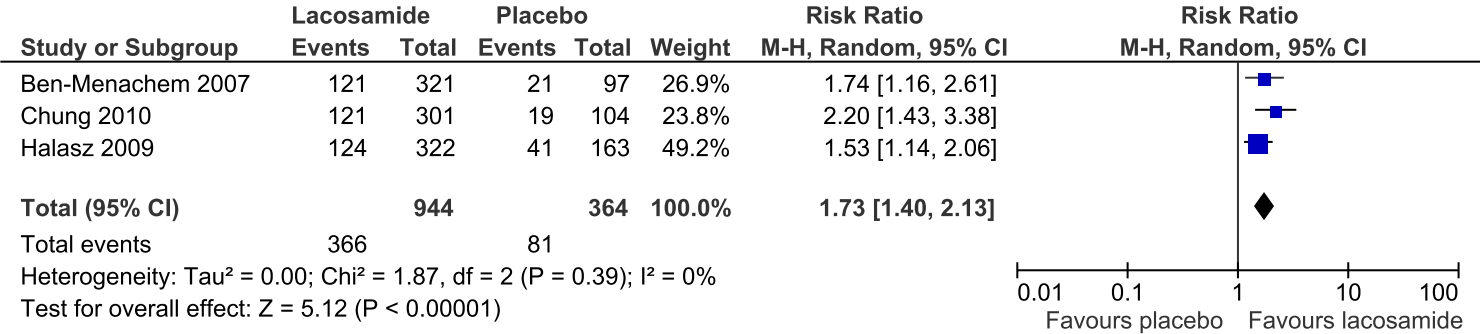

Supplement: Supplemental Information 6 [file peerj-01-114-s006.pdf]

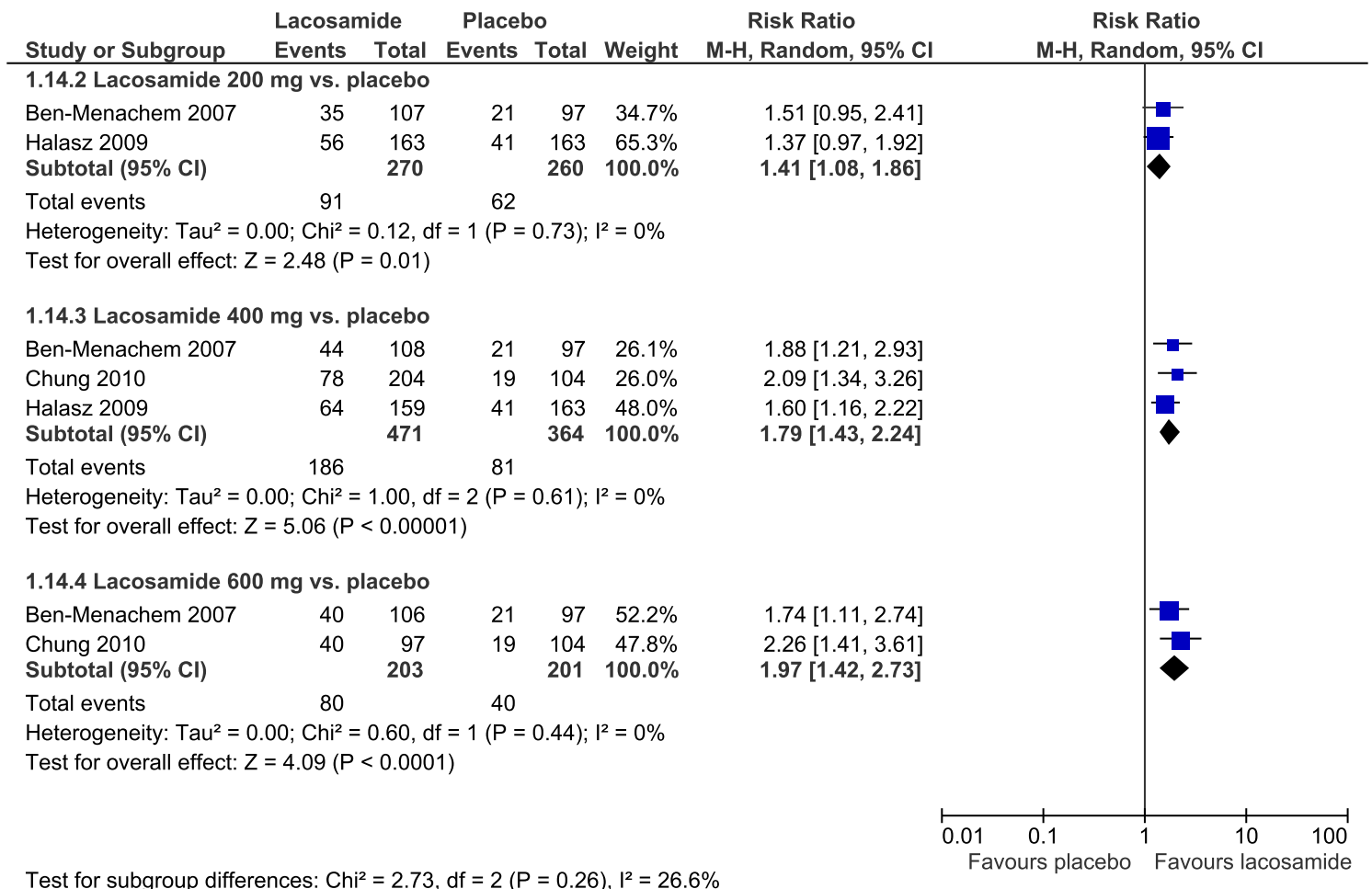

Supplement: Supplemental Information 7 [file peerj-01-114-s007.pdf]

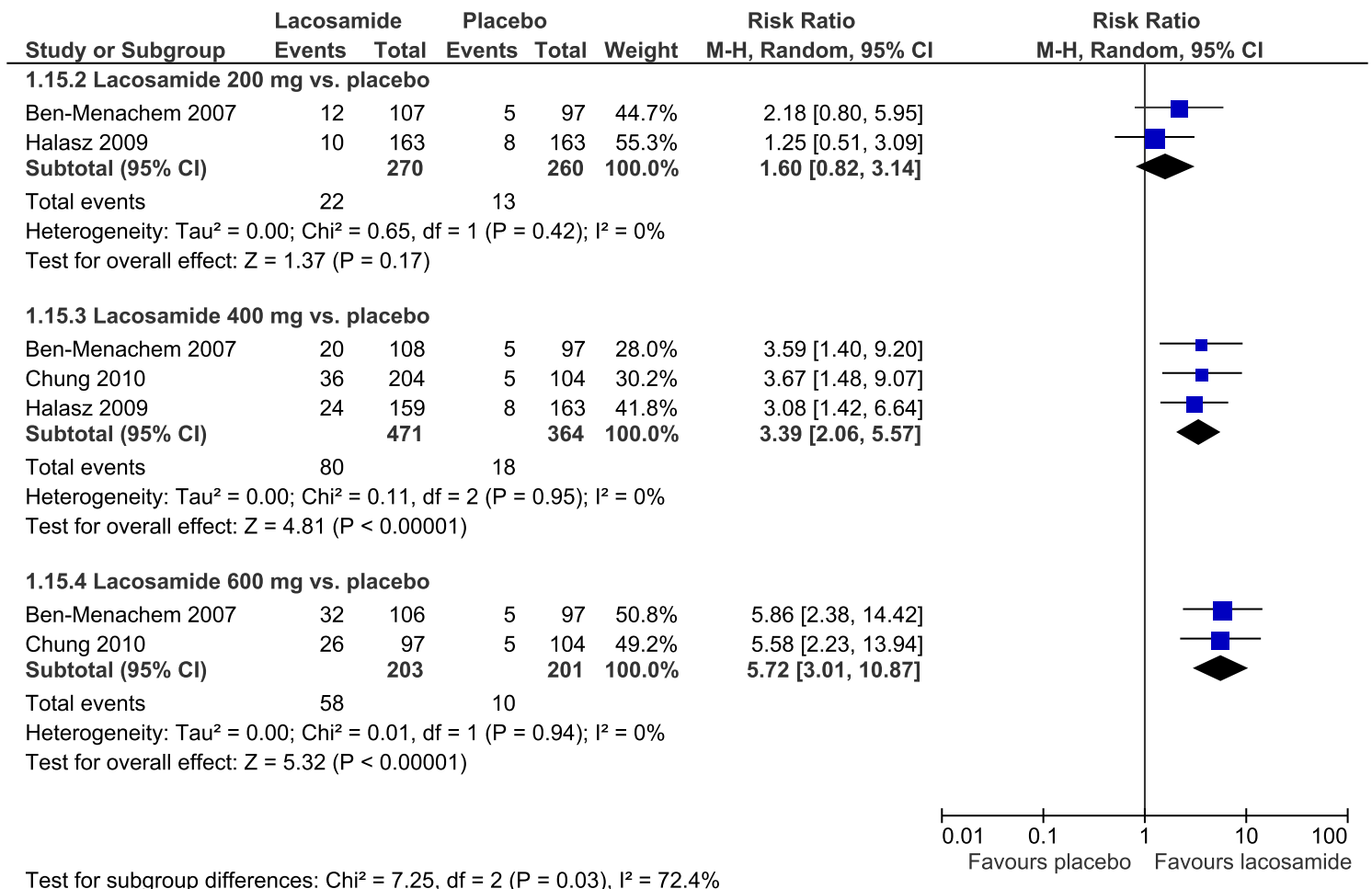

Supplement: Supplemental Information 8 [file peerj-01-114-s008.pdf]

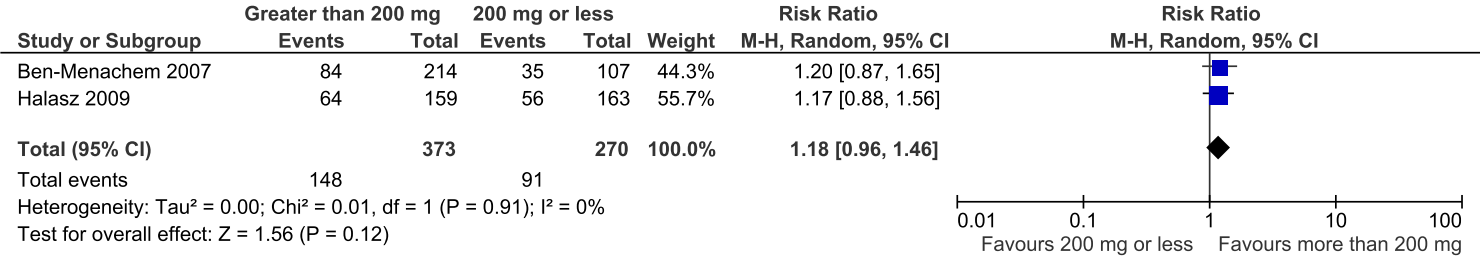

Supplement: Supplemental Information 9 [file peerj-01-114-s009.pdf]

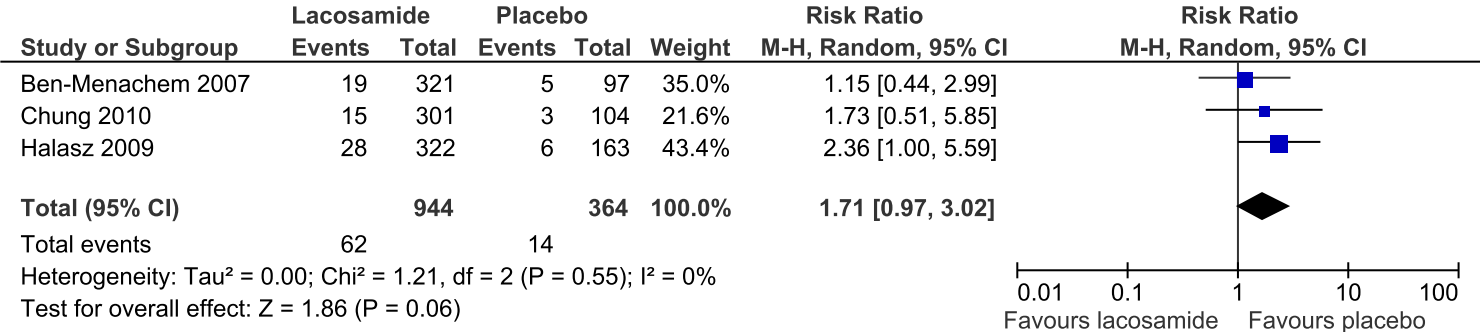

Supplement: Supplemental Information 15 [file peerj-01-114-s015.pdf]
