# Supplementary material for: Lacosamide adjunctive therapy for partial-onset seizures: a meta-analysis
Source: PeerJ. 2013 Aug 6;1:e114. doi: 10.7717/peerj.114 (PMC3740140; doi:10.7717/peerj.114)
Supplement: Table S1 [file peerj-01-114-s011.docx]

| **Reviewer:** | Choose an item. |
| --- | --- |
| **Title:** |  |
| **Last name of the first author & year published:** |  |
| **Language:** | Choose an item. Other: |

| **Criteria for exclusion (only need to have ONE criteria present to exclude):** | |
| --- | --- |
| Study design | ANY of the following  Observational study with a control group (e.g. cohort or case-control)  No independent control group (e.g. case report or case series)  Review (please keep systematic reviews separately for reference list checking)  Other type of publication (i.e. not a clinical study) |
| Population | ANY of the following  Patients without a confirmed diagnosis of partial epilepsy  Patients with and without partial epilepsy considered together (unless results for patients with partial epilepsy are reported separately) e.g. multiple epilepsy diagnoses in the same study  Pediatric patients (majority of patients less than 18 years old, unless reported as a subgroup) |
| Intervention | ANY of the following  Lacosamide in BOTH arms of the study, unless there are multiple arms comparing another antiepileptic or placebo against different doses of lacosamide  Single-dose or short-term lacosamide therapy for an acute seizure disorder |

| **Criteria for inclusion – see specific instructions below for which criteria are mandatory for inclusion** | |
| --- | --- |
| Study design | MUST be a Randomized controlled trial |
| Population | Adult patients (majority of patients over the age of 18), hospitalized or not, with a clinical diagnosis of partial-onset seizures (with or without secondary generalization) |
| Interventions in experimental or control groups | MUST include lacosamide (intravenous or oral or a combination of both)  **AND** ONE of the following as a comparator:  any other antiepileptic  placebo |
| Outcomes | ANY one of the following outcomes are reported  Change in seizure frequency from baseline  Responder rate at (proportion of patients with a 50% or greater reduction in seizure frequency in the treatment period compared to the pre-randomization baseline period)  Discontinuation of study drug due to adverse effects  Proportion of patients that were seizure-free during the treatment period  Adverse effects including: Ataxia, dizziness, fatigue, headache, nausea and somnolence  Serious adverse effects  Hospital admission  Length of hospital stay  Length of stay in a specialized epilepsy unit  Quality of life as reported by standardized scoring instruments (e.g. Quality of life inventory in epilepsy – QOLIE-31 scale, Seizure Severity Scale – SSS, Patient Global Impression of Change – PGIC scale)  Economic outcomes as captured by the search strategy (drug costs, hospitalization costs, caregiver costs, caregiver time) |

| **TO BE INCLUDED** |  |
| --- | --- |
| FURTHER ACTION REQUIRED | What action: |
| **TO BE EXCLUDED** REASON: | Non-randomized study design  Patients without a diagnosis of partial epilepsy  Pediatric patients  Lacosamide – single dose or short-term therapy  Outcomes – none of relevance to review/or clinically important  Other: |

Additional comments:
