# Supplementary material for: Lacosamide adjunctive therapy for partial-onset seizures: a meta-analysis
Source: PeerJ. 2013 Aug 6;1:e114. doi: 10.7717/peerj.114 (PMC3740140; doi:10.7717/peerj.114)
Supplement: Table S2 — Studies not included in the meta-analysis and reasons for exclusion. [file peerj-01-114-s012.docx]

| First Author & Publication Year | Reason for Exclusion |
| --- | --- |
| Abramowicz2009 (48) | Non-RCT |
| Benbadis2009 (49) | Non-RCT |
| Ben-Menachem2006 (50) | Non-RCT |
| Ben-Menachem2009a (51) | Non-RCT |
| Ben-Menachem2009b (52) | Non-RCT |
| Berggren2009 (53) | Non-RCT |
| Beydoun2009 (6) | Non-RCT |
| Beyenburg2010 (2) | Non-RCT |
| Biton2005 (54) | Lacosamide both arms |
| Biton2009 (55) | Non-RCT |
| Bonnaud2009 (56) | Non-RCT |
| Cada2009 (57) | Non-RCT |
| Chung 2010a (7) | Non-RCT |
| Chung2009b (58) | Non-RCT |
| Costa2011 (9) | Non-RCT |
| Davies2010 (59) | Non-RCT |
| deLaLoge2009 (60) | Non-RCT |
| Faught2010 (61) | Non-RCT |
| French2009a (62) | Non-RCT |
| French2009b (63) | Non-RCT |
| French2010 (64) | Non-RCT |
| Gil-Nagel2009 (65) | Non-RCT |
| Goldenberg2010 (66) | Non-RCT |
| Grellet 2010 (67) | Non-RCT |
| Harris2009 (68) | Non-RCT |
| Hebert2009 (69) | Non-RCT |
| Hovinga2003 (70) | Non-RCT |
| Husain2011 (38) | Non-RCT |
| Hussar2009 (71) | Non-RCT |
| Isojarvi2009 (72) | Non-RCT |
| Isojarvi2010 (73) | Non-RCT |
| Isojarvi2010b (74) | Non-RCT |
| Jatuzis2005 (35) | Duplicate publication |
| Jatuzis2006a (75) | Duplicate publication |
| Jatuzis2006b (75) | Duplicate publication |
| Kramer2009 (76) | Non-RCT |
| Mil’chakova2010 (77) | Non-RCT |
| Mucke 2003 (78) | Non-RCT |
| Perucca2008 (79) | Non-RCT |
| Prescrire Editorial Staff2009 (80) | Non-RCT |
| Rheims2010 (81) | Non-RCT |
| Rheims2011 (81) | Non-RCT |
| Richard2007 (82) | Non-RCT |
| Rosenfeld2005 (83) | Lacosamide both arms |
| Rosenfeld2008 (84) | Non-RCT |
| Rosenfeld2009a (85) | Non-RCT |
| Rosenfeld2009b (86) | Non-RCT |
| Rosenfeld2011 (39) | Non-RCT |
| Rosenfeld2011a (87) | Non-RCT |
| Rosenow2011 (40) | Non-RCT |
| Rudzinski2011 (88) | Non-RCT |
| Ryvlin2011 (10) | Non-RCT |
| Sake2010 (89) | Non-RCT |
| Sake2010b (90) | Non-RCT |
| Sake2010c (91) | Non-RCT |
| Saussele2008 (92) | Non-RCT |
| Schmidt2008 (93) | Non-RCT |
| Schmidt2010 (94) | Non-RCT |
| Schmitz2010 (95) | Non-RCT |
| Simoens2010 (43) | Non-RCT |
| Simoens2011 (8) | Non-RCT |
| Tabarki2009 (96) | Non-RCT |
| AES Conference Abstract (Jatsuzis 2005b) (35) | Duplicate publication |
| ECE abstracts #1 (Jatuzis 2006a) (35) | Non-RCT |
| ECE abstract #2 (Chung 2009b) (58) | Non-RCT |
| [NCT01190098](http://clinicaltrials.gov/ct2/show/NCT01190098) (97) | Ongoing trial |

Non RCT = non-randomized controlled trial
