# Supplementary material for: Lacosamide adjunctive therapy for partial-onset seizures: a meta-analysis
Source: PeerJ. 2013 Aug 6;1:e114. doi: 10.7717/peerj.114 (PMC3740140; doi:10.7717/peerj.114)
Supplement: Table S3 — Provided in Canadian $ per day. [file peerj-01-114-s013.docx]

| Drug Generic Name | Dosage Regimen | Cost per day ($CAD) |
| --- | --- | --- |
| Carbamazepine | 400 mg QID  400 mg CR BID | $0.60  $0.37 |
| Divalproex | 500 mg BID | $0.52 |
| Ethosuximide | 500 mg BID | $1.35 |
| Gapabentin | 600 mg TID | $1.55 |
| Lacosamide | 200 mg BID | $10.64 |
| Lamotrigine | 100 mg BID | $0.75 |
| Levetiracetam | 500 mg BID | $3.00 |
| Oxcarbazepine | 600 mg BID | $5.23 |
| Phenytoin | 300 mg HS | $0.22 |
| Topiramate | 100 mg BID | $1.19 |
| Valproic acid | 500 mg BID | $0.83 |
| Vigabatrin | 1000 mg BID | $3.64 |

*Costs based on Ontario Drug Benefit (ODB) Formulary (54), Common Drug Review (50) and RxFiles(49) for generic products where available.

**None of the above “usual doses” (49) imply therapeutic or clinical equivalency

AED = antiepileptic drugs
